# Development of a digital cloud imaging platform based on a “pay-for-performance” model: an empirical study on cost control and resource conservation

**DOI:** 10.3389/fpubh.2026.1904169

**Published:** 2026-09-30

**Authors:** Xingbo Long, Min Huang, Qing Ye, Xu Liu

**Affiliations:** 1 Department of Medical and Information Engineering, Deyang People’s Hospital, Deyang, Sichuan, China; 2 Department of Quality Improvement, Deyang People’s Hospital, Deyang, Sichuan, China

**Keywords:** cloud-based medical images, digital imaging, healthcare cost control, hospital management, interrupted time series analysis, smart healthcare

## Abstract

**Introduction:**

Traditional physical film-based imaging services impose a heavy financial and operational burden on public hospitals. This study aims to evaluate the actual effectiveness of a digital cloud imaging platform—deployed under a model where “service providers make upfront investments and hospitals pay based on performance”—in terms of controlling medical costs, optimizing clinical workflows, and enhancing the patient experience, thereby providing a replicable management pathway and empirical evidence for the digital transformation of imaging services.

**Methods:**

A single-center, quasi-experimental before-and-after study design was adopted, supplemented by interrupted time series (ITS) analysis as the primary method for causal inference, with Deyang People’s Hospital serving as the study site. Prior to project implementation, the study included data from 160,183 eligible imaging examinations; after implementation, it included data from 295,940 eligible imaging examinations. The platform was built on a hybrid cloud architecture. Through collaboration with a third-party technology provider, the hospital was able to deploy the system without upfront capital expenditures, replacing traditional physical films with “cloud films.” Operational data was collected from the hospital’s PACS system and the administrative backend of the digital cloud imaging service platform both before the platform’s launch (January 1, 2022–December 31, 2022) and after (August 12, 2024–August 11, 2025). Descriptive statistical methods, the Mann–Whitney U test, and interrupted time series analysis to compare differences in data before and after project implementation, thereby evaluating the system’s actual effectiveness.

**Results:**

Within 12 months of the platform’s operation, the hospital achieved net savings of 4,159,500 yuan on film-related consumables; the median time from examination to reporting for physicians was reduced from 6.86 h to 2.41 h (*p* < 0.001), a decrease of approximately 64.9%, resulting in a significant improvement in work efficiency. Patients experienced “zero wait time” for film retrieval, shortening their total visit duration; interrupted time series analysis showed that patient satisfaction exhibited a sustained quarterly upward trend following the intervention, increasing by an average of 0.46 percentage points per quarter (*P* < 0.001), though no immediate jump in satisfaction was detected at the time of intervention implementation (*p* = 0.440).

**Discussion:**

Within the framework of this study’s single-center, quasi-experimental design, a temporal association was observed between the “service provider upfront investment, pay-for-performance” model of the digital cloud imaging platform and reductions in film and consumable costs, improvements in physicians’ image-reading efficiency, and enhancements in patient satisfaction. However, due to limitations in the study design, further evidence is needed to confirm the causality of these associations. The long-term financial sustainability and cross-institutional scalability of this model require validation through longer-term follow-up and multicenter studies.

## 1 Introduction

With the rapid development of tomographic imaging technologies, particularly the widespread adoption of computed tomography (CT) and magnetic resonance imaging (MRI), the volume of medical imaging examinations worldwide continues to rise, placing an increasingly heavy burden on radiology workflows that rely on traditional physical film. In high-income countries such as those in Europe and the United States, medical institutions have largely completed the transition from physical film to Picture Archiving and Communication Systems (PACS) based on the Digital Imaging and Communications in Medicine (DICOM) standard, achieving filmless digital image management (1). However, in many low- and middle-income countries or regions, including China, a significant proportion of public hospitals still rely on traditional plastic film as the primary medium for image delivery (2).

China’s medical film market is massive; Class A tertiary hospitals alone spend up to several million yuan annually on film procurement, in addition to incurring hidden costs such as maintenance of printing equipment, expansion of storage capacity, and personnel expenses for film management (3–5). The traditional physical film model faces three prominent challenges: First, direct economic costs and hidden operational costs remain high; second, the information capacity of a single film is limited, making it difficult to meet the diagnostic needs of the massive image data generated by modern multi-slice CT or volumetric MRI scans. Patients must wait in line for prints, and films are prone to loss or damage when seeking care at different hospitals, leading to a persistently high rate of repeat examinations (6–8); Third, polyester-based silver halide film has a long natural degradation cycle, and its disposal poses environmental pollution risks (9); at the same time, it is difficult to meet the legal requirements for retaining outpatient medical records for 15 years and inpatient medical records for 30 years (10).

To promote the transformation and upgrading of imaging services, the Chinese government has issued a series of policy documents in recent years (11–14), calling for accelerated progress in the interoperability of imaging data and incorporating digital transformation and cost reduction with efficiency improvements into the core agenda for the development of public hospitals. These policy initiatives have sparked widespread interest in cloud-based digital imaging platforms (commonly referred to as “cloud films”). Cloud film platforms store complete DICOM-format image data on remote cloud servers, enabling secure, real-time, on-demand access to imaging data for patients and clinicians via mobile devices or web browsers (15, 16).

International experience has demonstrated that the transition from physical film to fully digital imaging workflows yields significant cost savings and quality improvements. After deploying a clinical information system integrated with RIS/PACS across its network of 40 hospitals, Mexico’s Social Security Institute (ISSSTE) successfully eliminated its reliance on traditional radiographic film, achieving significant cost savings and environmental benefits (1). Numerous studies in the United States, Europe, and South Korea have also reported that the implementation of PACS systems can reduce departmental film costs by 40–70% while shortening report turnaround times (17, 18).

However, the applicability of the aforementioned international experiences within the context of China’s public hospitals is constrained by differences in healthcare financing structures and regulatory frameworks, as well as the unique dilemma of high patient volumes coupled with limited budgets. In domestic practice, early cloud film projects often adopted a value-added service model requiring “voluntary patient payment for access,” resulting in low usage rates and frequent patient complaints, and failing to effectively achieve the dual objectives of cost control and resource conservation alongside patient convenience (6). Although recent literature has reported on the implementation experiences of cloud-based radiology systems in some single-center settings, these studies are primarily descriptive in nature, lacking rigorous before-and-after comparative designs, and rarely provide systematic quantification of multi-dimensional indicators such as economic benefits, process efficiency, and patient experience (15, 16). Furthermore, practical challenges such as high initial investment costs, significant operational and maintenance pressures, and unclear business models have further hindered the large-scale adoption of digital cloud imaging platforms in public hospitals.

It is worth noting that no studies have yet systematically evaluated the “service provider upfront investment, pay-for-performance” cooperation model. It differs significantly from existing SaaS (Software as a Service), managed services, and PPP (Public-Private Partnership) models: Under the SaaS model, hospitals typically pay fixed software subscription fees on a regular basis, regardless of usage; in the managed services model, the service provider handles system operation and maintenance, but the hospital still bears the cost of hardware procurement; the PPP model involves complex equity partnerships and long-term contractual obligations. In contrast, the core features of this model are: (1) Zero capital expenditures—hospitals do not need to purchase any hardware or software licenses, as the technology provider bears the upfront capital investment; (2) Purely variable costs—service fees are based entirely on the actual usage of cloud-based medical images (billed per patient visit), with no fixed fees; returns are earned based on actual performance, directly addressing the core challenge of rigid budget constraints faced by public hospitals; (3) Complete risk transfer—the service provider assumes all market risks associated with the inability to recoup upfront investments. This is the first systematic report on this model in the field of medical imaging.

To address the aforementioned research gap, Deyang People’s Hospital took the lead in 2022 by introducing a “service provider upfront investment, pay-for-performance” partnership model, establishing a full-process digital cloud imaging service platform that covers in-hospital diagnosis and treatment, regional collaboration, and patient services. This study employed a quasi-experimental before-and-after design, supplemented by medium-term time-series analysis, to systematically evaluate the platform’s implementation outcomes across the following three dimensions: (1) control of medical consumables costs; (2) optimization of imaging service process efficiency; and (3) improvement of patient experience and satisfaction. The study aims to provide an evidence-based, replicable, and scalable management model and scientific reference for the digital transformation of imaging services in China’s public hospitals.

## 2 Subjects and methods

### 2.1 Study design

This study employed a single-center, quasi-experimental pre-post design, using the construction project of the Digital Cloud Imaging Service Platform at Deyang People’s Hospital as the intervention, with outpatients and inpatients undergoing imaging examinations in the hospital’s Department of Radiology serving as the study subjects. The study period encompassed the baseline phase (prior to project implementation, January 1, 2022, to August 11, 2024), the intervention phase (the platform’s trial operation and gradual rollout), and the phase following full-scale implementation.

To enhance the rigor of causal inference, this study employed interrupted time series (ITS) analysis as the primary analytical method. ITS utilizes the timing of intervention implementation as a “natural break” and estimates the intervention effect by comparing changes in the slopes and intercepts of outcome measures before and after the intervention. Compared with simple before-and-after comparison designs, ITS effectively controls for confounding factors such as long-term trends and seasonal fluctuations that existed prior to the intervention. In real-world health services research, because the digital cloud imaging service platform was implemented comprehensively at the hospital level, it was difficult to establish a contemporaneous control group within the same institution that had not received the intervention, and it was also difficult to find a control hospital with perfectly matched characteristics in all respects. In this context, interrupted time series analysis is recognized as one of the most feasible quasi-experimental design methods and has been widely used to evaluate the effects of policy interventions, system reforms, and the introduction of new technologies.

### 2.2 Study population

This study included all outpatients and inpatients who underwent CT, MR, or DR imaging examinations at the Department of Radiology of Deyang People’s Hospital both before the project’s implementation (January 1, 2022–December 31, 2022) and after its launch (August 12, 2024–August 11, 2025). Inclusion criteria: (1) Age ≥ 18 years; (2) Imaging examinations were performed at Deyang People’s Hospital; (3) Examination data were complete and traceable. Exclusion criteria: (1) Patients in the emergency green channel (who, due to the special nature of their procedures, did not follow the standard film delivery process); (2) Consultation cases transferred from other hospitals (whose imaging data were not generated by this hospital’s PACS system). During the study period, a total of 160,183 eligible imaging examinations were included prior to project implementation, and a total of 295,940 eligible imaging examinations were included after project implementation (231,086 CT/MR examinations in Phase I, and 64,854 examinations across the entire Radiology Department in Phase II).

Supplementary note on the difference in sample size before and after the intervention: The pre-intervention sample (*n* = 160,183) consists of data from the entire year of 2022 (12 months); the post-intervention sample (*n* = 295,940) consists of data from August 2024 to August 2025 (13 months), and the scope of coverage was expanded from CT/MR in Phase 1 to the entire radiology department in Phase 2 (including DR, MG, and other examination items); therefore, the total number of examinations is higher than that in the pre-intervention period. Within the ITS analytical framework, changes in sample size over time do not in themselves constitute a methodological obstacle—ITS focuses on trends in outcome measures over time series, rather than direct comparisons of sample sizes between the pre- and post-intervention periods. Changes in sample size at various time points (such as natural growth in the volume of examinations) are accounted for by the time-trend term in the model and do not introduce substantial bias in the estimation of the intervention effect.

### 2.3 Ethical review

This study constitutes a retrospective systematic evaluation and analysis based on hospital operational data. It does not involve any clinical trials involving human or animal subjects, nor does it collect or utilize any personally identifiable information (including personal details of patients, medical staff, or other participants). This retrospective study was conducted in accordance with the Declaration of Helsinki. The study protocol was reviewed and approved by the Ethics Committee of Deyang People’s Hospital (Ethics Review Number: 2024–03-004-K01). Given the retrospective nature of the study and the use of fully anonymized data extracted from the hospital’s PACS system and the digital cloud imaging platform management backend, which contained no personally identifiable information, the Ethics Committee waived the requirement for individual informed consent. All methods were carried out in accordance with relevant guidelines and regulations.

### 2.4 Technical architecture design

A digital cloud imaging service platform was built using a hybrid cloud architecture (19), as shown in Figure 1. It consists of a three-tier architecture—the on-premises layer, the cloud layer, and the collaboration layer—that balances data security with service scalability. This design enables both efficient processing of on-premises data and the elastic storage of off-site data. While ensuring data security and access speed, it effectively overcomes the scalability limitations of traditional PACS systems.

**Figure 1:**
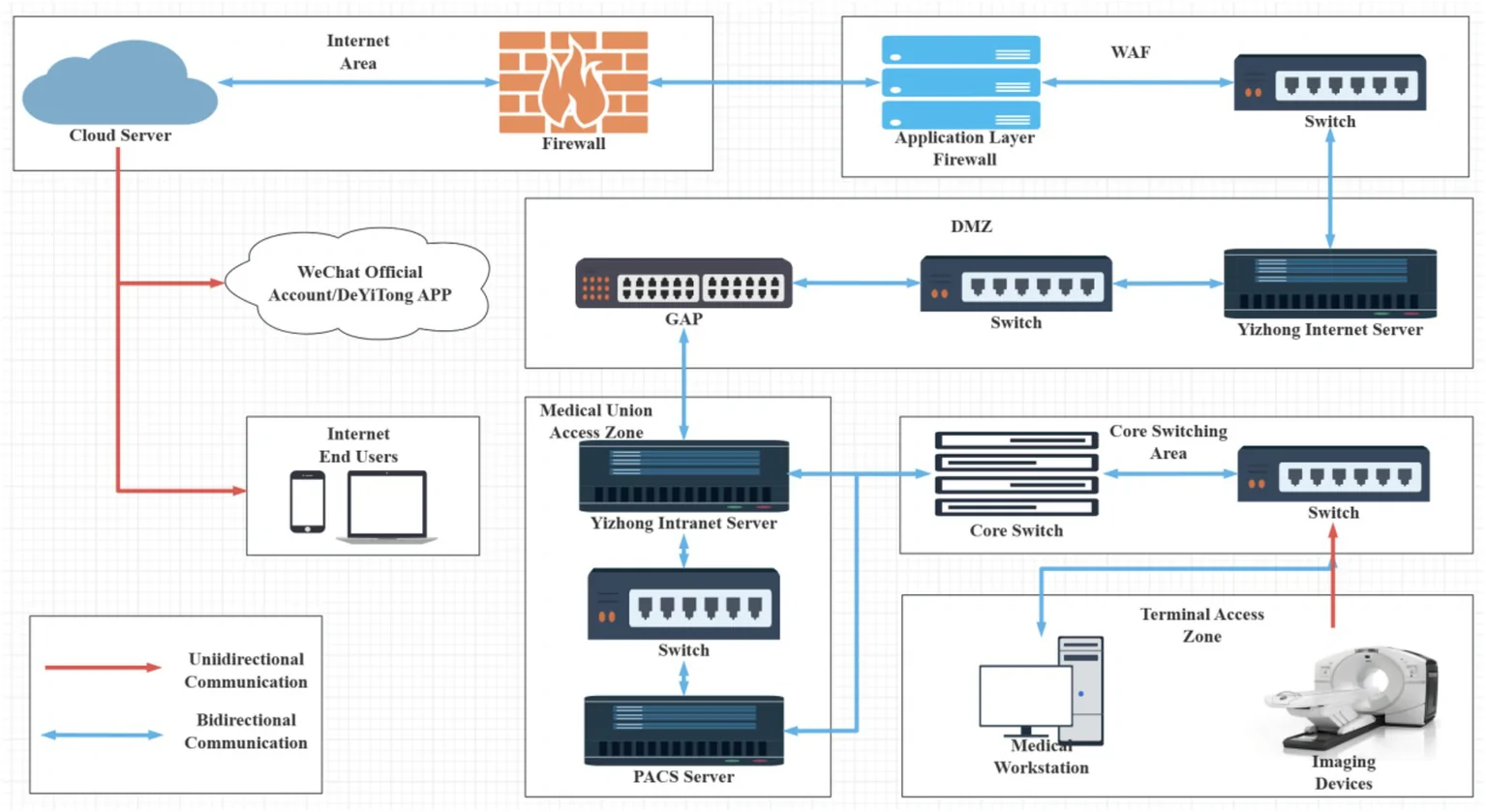
Hybrid cloud technical architecture for the digital cloud imaging service platform.

The on-premises layer is used to deploy local gateways and application servers, enabling functions such as real-time image data acquisition and near-line storage. It is deeply integrated with the hospital’s HIS, electronic medical record system, and PACS to ensure the interoperability of patient diagnosis and treatment data, thereby guaranteeing the continuity of on-premises diagnostic and treatment processes. The cloud layer provides exabyte-scale elastic storage and long-term data disaster recovery through Medical Imaging Object Storage (MOS) cloud services. The collaboration layer enables physicians to view images on the go, conduct remote consultations, and allows patients to access their cloud-based imaging records via terminals such as internet workstations, WeChat official accounts, and hospital apps.

Regarding the definition of software innovation, it should be clarified that the core contribution of this study lies not in original innovation at the underlying software level, but rather in integrated innovation and model innovation. Specifically: (1) Architectural integration innovation. The team developed an in-hospital data gateway middleware that automates the extraction, de-identification, compression, and encrypted upload of DICOM data from the PACS system, as well as a module for anonymizing the association between patient identity information and imaging data; (2) Operational model innovation: The feasibility of the “service provider upfront investment, pay-for-performance” model was validated. This definition aligns with the paradigm of implementation science, which involves evaluating the effectiveness of new organizational and delivery models for an existing technology in a real-world setting.

### 2.5 Image storage architecture and data flow

To clearly define the roles and relationships of various storage media before and after project implementation, the image data storage architecture is described below.

Before Implementation (Traditional Model): All raw DICOM data generated from imaging examinations is stored in the hospital’s on-premises PACS system (online storage + nearline storage), with the storage period limited by local storage capacity. Physical plastic film is a medium produced by technicians who select key image sequences from the DICOM data, arrange them, and print them; it is provided solely to patients for presentation during medical visits or referrals and does not serve as the hospital’s official archival medium. The hospital’s official imaging records consist of the raw DICOM data stored in the PACS system.

Post-Implementation (Digital Cloud Imaging Model): Following the platform’s launch, the imaging data storage architecture was adjusted to a “local + cloud” dual-track system. (1) PACS System (Official Archive) — All raw DICOM data continues to be stored in full within the hospital’s on-premises PACS system, serving as the official archive of legally mandated medical records; (2) Cloud Storage (Disaster Recovery and Convenient Access) — The raw DICOM data from the PACS is automatically synchronized via an on-premises gateway to the cloud-based MOS storage, serving as an off-site disaster recovery copy. It also functions as the data source for patient access and mobile image review by physicians, with storage periods strictly configured in accordance with national standards: 15 years for outpatient records and 30 years for inpatient records; (3) Physical Film (On-Demand Printing)—No longer provided as a standard deliverable; patients may request printing at their own expense only when there are special needs. The relationship among these three components can be summarized as follows: PACS serves as the legally mandated official archive, the cloud serves as the disaster recovery and convenient access layer, and physical film is an optional, on-demand add-on service.

### 2.6 Data security and privacy protection

To ensure the security and compliance of patient imaging data throughout its entire lifecycle—including transmission, storage, and retrieval—the platform has established the following protection system: (1) Transmission Encryption—All imaging data exported from the hospital’s PACS system to the cloud is transmitted via end-to-end encryption using the TLS 1.3 protocol, and all retrieval requests are initiated through a secure HTTPS channel. (2) Identity Authentication and Access Control—On the physician side, the hospital’s unified identity authentication system (LDAP/SSO) is used for two-factor authentication; on the patient side, identity verification is performed via mobile phone number and a real-time verification code. The system employs a role-based access control (RBAC) model. (3) Operational Audit Logs—Full-link logging is performed for all image viewing, downloading, and sharing operations, with logs retained for no less than 3 years. (4) QR Code Security—The QR codes on examination reports utilize time-sensitive dynamic token technology and are bound to the patient’s examination number and mobile phone number to prevent misuse across devices. (5) Tiered Data Storage and Privacy Isolation—Patient identity information and image pixel data are physically separated during storage and linked via irreversible anonymized identifiers. (6) Third-Party Service Provider Responsibilities — In accordance with the Service Level Agreement (SLA), service providers must hold Level 3 certification under the National Information Security Graded Protection System. They may not use data for any purpose not specified in the contract without the hospital’s authorization. In the event of a security incident, they must notify the hospital within 2 h and initiate an emergency response.

### 2.7 Implementation path

To achieve the project’s objectives, an iterative and incremental project management approach (20) has been adopted, consisting of a five-phase progressive implementation plan: “Research and Planning—Development and Integration—Pilot Validation—Full-Scale Rollout—Optimization and Enhancement.” Compared to traditional project management methods, this approach emphasizes adaptability, flexibility, and rapid responsiveness to change, ensuring the smooth implementation of the digital cloud imaging service platform, guaranteeing the quality of the project, and better aligning it with the practical needs of hospitals and patients. The project implementation timeline is shown in Figure 2.

**Figure 2:**
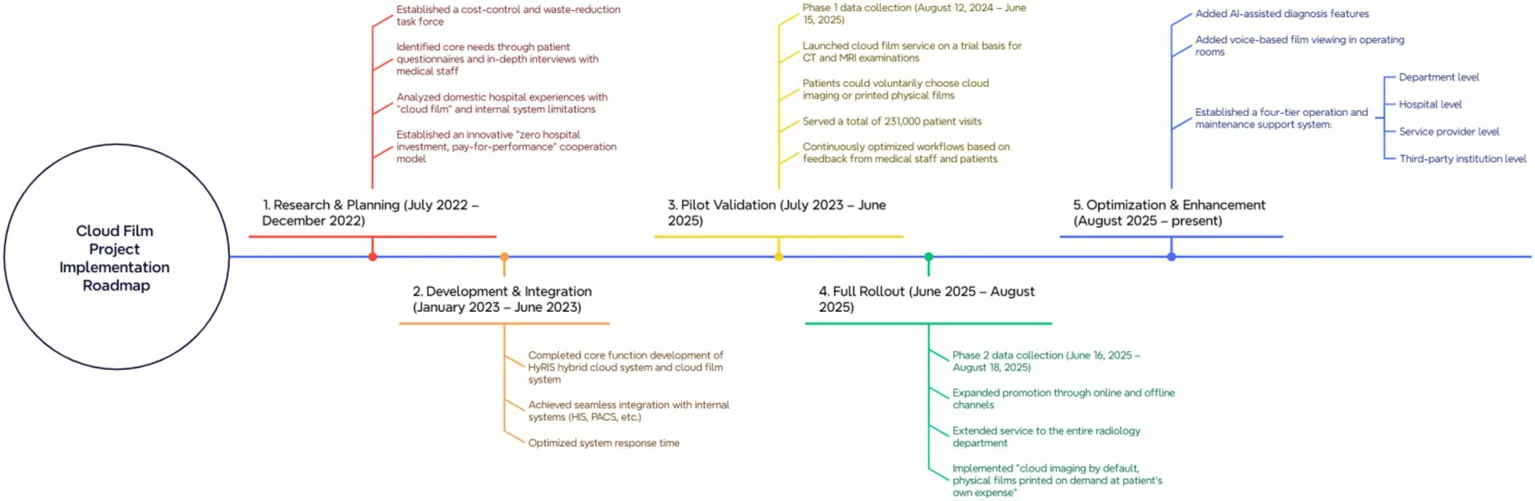
Project implementation timeline.

The key implementation details directly related to the evaluation of this study’s effectiveness are as follows.

Workflow Changes: Prior to implementation, after completing an imaging exam, patients had to wait in line at a self-service kiosk to print physical films (average wait time: 60 min), and technologists had to select key image sequences for each patient and arrange them for printing. After implementation, DICOM data is automatically uploaded to the cloud to generate cloud films upon completion of imaging exams; patients can view the images instantly on their mobile phones by scanning a QR code or receiving a push notification from the official WeChat account. Technicians are no longer responsible for film printing and can reallocate their time to image quality control.

User Training: During the full-scale rollout phase (January–July 2024), the research team organized system training sessions tailored by job role and conducted in batches. The training followed a “demonstration + hands-on practice + assessment” model, and all trainees were required to pass an operational assessment before being cleared to work.

Patient Guidance: During the initial launch phase of the platform (August–October 2024), the hospital stationed volunteers in the radiology department’s waiting area to provide on-site guidance to patients on how to scan the QR code and access their images for the first time, and distributed simple user guides.

### 2.8 Key performance indicators and data analysis

#### 2.8.1 Evaluation metrics

An evaluation metric system is established based on three dimensions: economic benefits, operational efficiency, and service quality.

Economic Benefit Metrics: Total number of examinations at each stage (intervention transition period, full-scale rollout period); number of digital cloud imaging sessions; number of traditional film prints; projected film expenditure (calculated based on historical usage); actual film expenditure; cloud imaging platform service fees; and net savings. Net Savings = Projected Film Expenditures Under the Traditional Model − (Actual Film Expenditures + Cloud Imaging Service Fees). Here, “Projected Film Expenditures Under the Traditional Model” is calculated based on the average film print volume for each examination type during the 12 months prior to project implementation (average of 1.35 sheets per person for CT/MR and 0.67 sheets per person for DR) multiplied by the unit purchase price of film during the same period (16.5 yuan per sheet).

Operational Efficiency Metric: The time elapsed from the completion of a patient’s examination to the doctor’s completion of image review and report generation (referred to as “time from exam to report”); data is sourced from the timestamps in the PACS system’s operation logs.

Service Quality Indicators: Patient satisfaction.

#### 2.8.2 Data sources and time frame

Data on the number of patient visits, cloud imaging sessions, number of film prints, and expenses: Sourced from automatic logs and billing records in the hospital’s PACS system and the digital cloud imaging service platform’s administrative backend. Data time frame: August 12, 2024, to August 11, 2025.

Time from Examination to Report: Calculated by extracting the examination and reporting timestamps for each examination item from the PACS system operation logs and the reporting system, comparing the periods before project implementation (January 1, 2022 – December 31, 2022) and after system launch (August 12, 2024 – August 11, 2025).

Patient Satisfaction: Data were derived from the summary reports of satisfaction surveys conducted quarterly by a third-party professional organization commissioned by the hospital’s Quality Improvement Department. The surveys were independently administered by the third-party organization, and data collection was conducted separately from this research team. The reports provided aggregated statistical results; this study extracted only the satisfaction scores directly related to “radiology report turnaround time.” This metric was consistently defined throughout the survey period, enabling longitudinal comparisons before and after the intervention. The data time range is from January 1, 2022, to September 30, 2025. Due to practical constraints regarding data accessibility and budgetary limitations, this study did not retrospectively collect raw survey data at the individual level, nor did it conduct a separate, independent patient satisfaction survey; these limitations are discussed in the Discussion section.

During this study, no human subjects were directly recruited, contacted, or intervened with, nor was any personally identifiable health information accessed.

#### 2.8.3 Strategies for controlling confounding factors

To control for temporal confounding factors commonly found in non-randomized before-and-after designs, this study adopted the following measures: (1) Long-term trend control—using the time-trend term in the ITS analysis to account for factors that change linearly over time, such as the natural increase in the volume of examinations and changes in film prices; (2) Seasonal control—incorporating month-specific dummy variables into the ITS model to account for monthly cyclical fluctuations in the volume of examinations; (3) Policy Change Note—During the study period, the National Healthcare Security Administration issued the *Guidelines for the Establishment of Pricing Items for Radiological Examination Services (Trial)*, stipulating that patients must purchase X-ray film out-of-pocket as needed. This policy change occurred during Phase 2 (full radiology department rollout) and may have had an independent effect on the hospital’s film expenditures. In our presentation of results, we clearly distinguished the economic benefit data for Phase 1 (prior to the policy change) and Phase 2 (after the policy change), and in the discussion section, we specifically addressed the impact of this policy change on the interpretation of the results.

#### 2.8.4 Statistical analysis methods

Continuous variables were described using the mean ± standard deviation or the median (interquartile range), while categorical variables were described using frequencies and percentages. Since the data did not follow a normal distribution (as confirmed by the Shapiro–Wilk test), the Mann–Whitney *U* test and interrupted time series analysis were used to compare differences in the data before and after project implementation. The ITS model was estimated using ordinary least squares (OLS), with autocorrelation and heteroscedasticity adjusted using Newey–West standard errors (first-order lag). Data analysis was performed using SPSS 27.0 and Python. All tests were two-sided, and a *p*-value < 0.05 was considered statistically significant. In addition to *p*-values, key effects were reported with both 95% confidence intervals and effect sizes (Cohen’s *d*).

## 3 Research findings

### 3.1 Cost control and energy conservation results

#### 3.1.1 Analysis of direct economic benefits

Since its launch, the project has achieved significant results in cost control and resource conservation. The use of physical plastic film—one of the hospital’s lowest-value consumables with the highest procurement value—has dropped significantly. From August 12, 2024, to August 11, 2025, the project generated cumulative net savings of 4,159,491.79 yuan in consumable-related expenses for the hospital.

During Phase 1 (August 12, 2024, to June 15, 2025, with CT/MR as the pilot), there were a total of 231,086 CT/MR examinations, of which 225,528 (97.6%) opted for cloud imaging. During Phase 1, both physical film and cloud film were available. The hospital fully respected patient preferences, allowing patients to choose between physical film and cloud film based on their individual needs. The trend chart for the monthly cloud film adoption rate (defined as the percentage of patients choosing cloud film when both options are available) is shown in Figure 3. As shown in the figure, the cloud film adoption rate exhibited a steady upward trend during Phase 1, rising from 95.52% at the project’s launch to 99.01%—an increase of 3.49 percentage points. This indicates that digital cloud imaging services are significantly preferred by patients over traditional plastic film.

**Figure 3:**
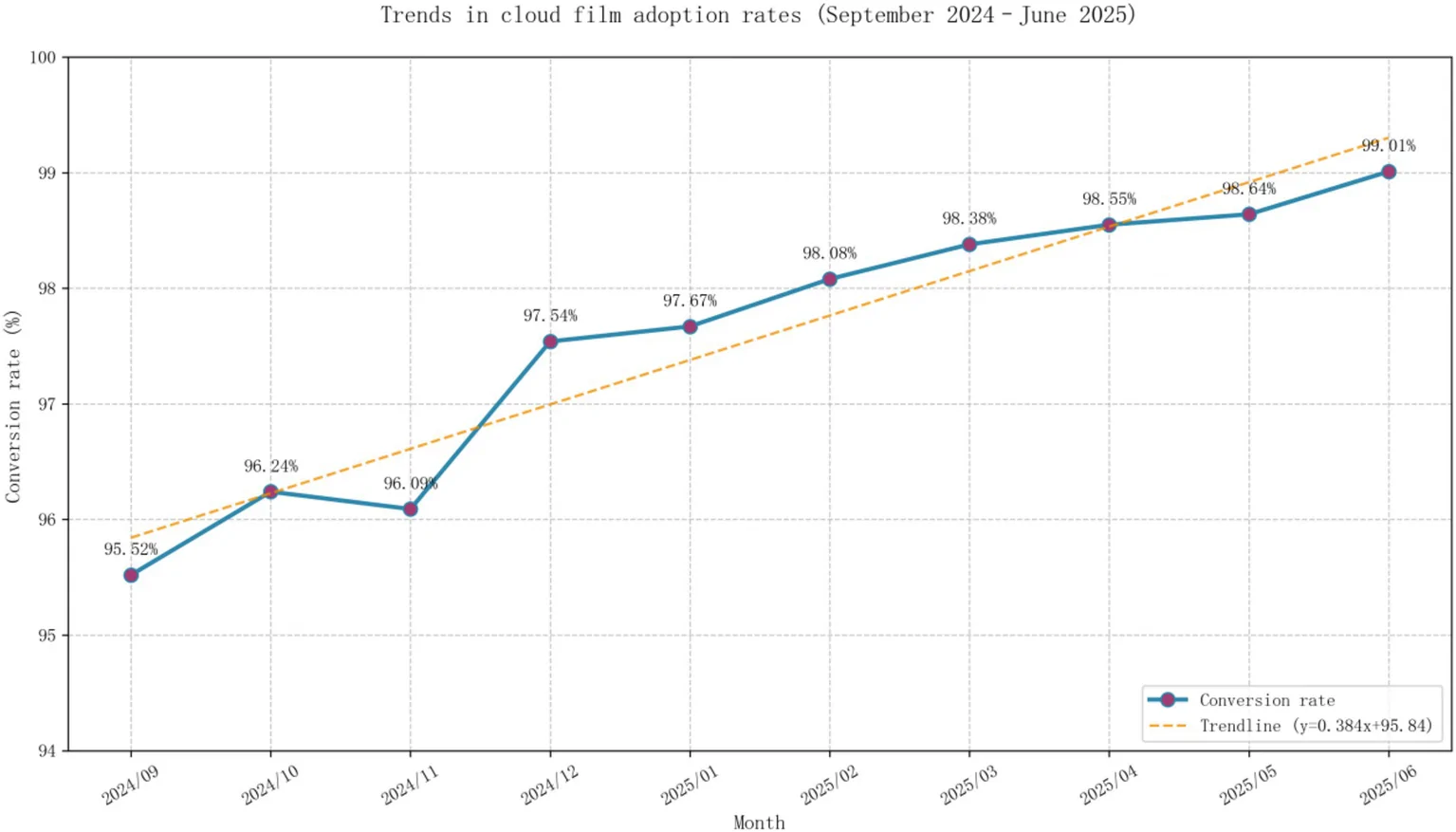
Trend chart for cloud film application conversion rates.

Based on historical data from previous years: the average number of films printed per person for CT/MR is approximately 1.35, and for DR, approximately 0.67. If the hospital’s film procurement price is calculated at a unit price of 16.5 yuan per sheet, and using the traditional model, the hospital would be projected to spend approximately 5.1474 million yuan on film costs for these examinations. In actual operation, film expenses were reduced to 278,100 yuan, while imaging cloud service fees amounted to 1,578,700 yuan, resulting in a net savings of 3,290,700 yuan in consumable-related costs for the hospital.

During the second phase (June 16, 2025, to August 11, 2025), digital cloud imaging services were expanded to cover all radiology examination items, with a total of 64,854 patient visits and a cloud imaging utilization rate of 100%. Based on projections using the traditional model, these examinations would have incurred film costs of approximately 1.2515 million yuan. Following the release of policy documents by the central government and Sichuan Province regarding the standardization of pricing for radiological examination services (21, 22), film is now purchased and printed at the patient’s own expense, resulting in zero actual film procurement costs for the hospital. After deducting the imaging cloud service fee of 382,700 yuan, the hospital achieved a net savings of 868,800 yuan in consumables procurement costs during this phase.

A detailed economic benefit analysis before and after project implementation is shown in Table 1, and the cost-saving results are illustrated in Figure 4.

**Table 1:** Analysis of economic benefits before and after project implementation (August 12, 2024 – August 11, 2025).

Phase	Project	Film expenses under the traditional model (CNY)	Actual film expenses (CNY)	Image cloud service fee (CNY)	Net savings (CNY)
Phase 1	CT/MRI scans	5,147,440.65	278,058.00	1,578,696.00	3,290,686.65
Phase 2	Full-body X-ray examination	1,251,537.88	0.00	382,732.74	868,805.14
Total		6,398,978.53	278,058.00	1,961,428.74	4,159,491.79

**Figure 4:**
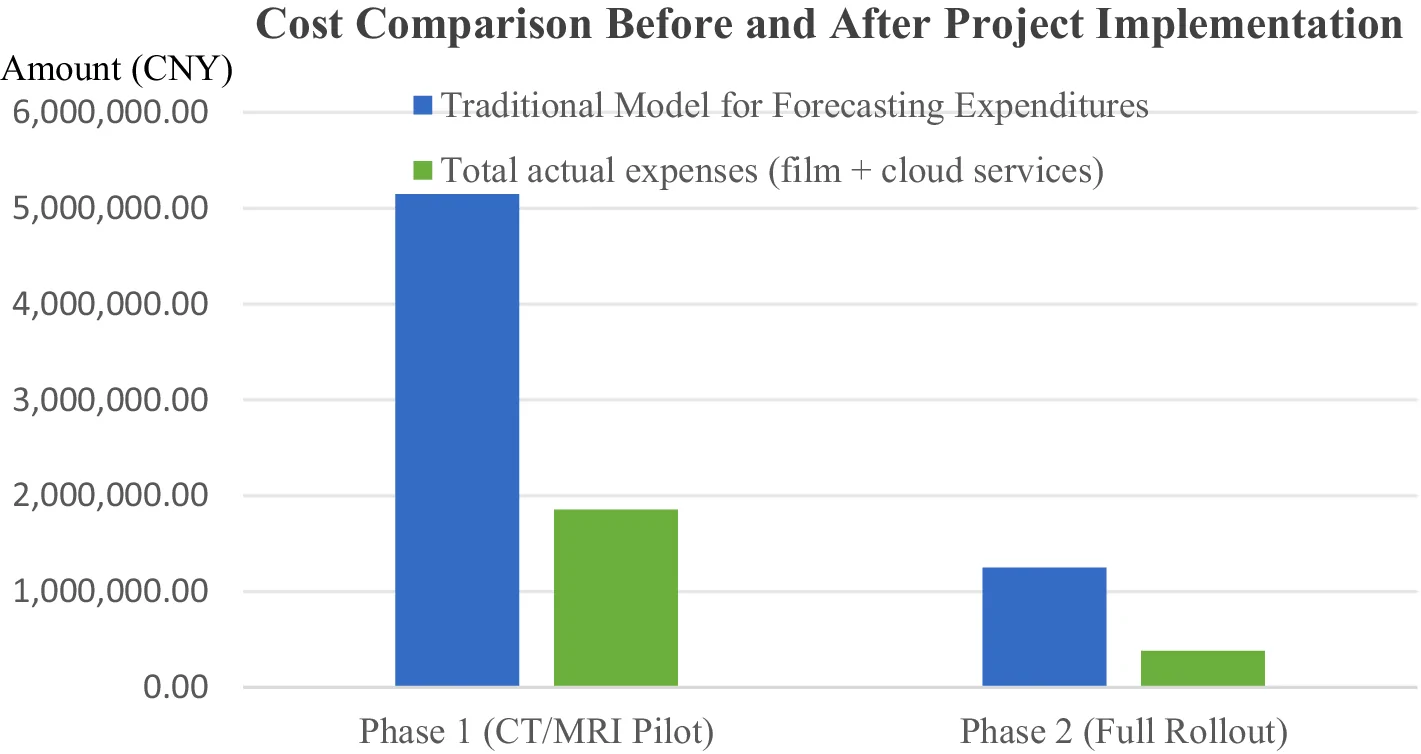
Cost comparison analysis before and after project implementation.

Assumptions and Sensitivity Analysis for Economic Benefit Calculations. The calculation of film expenditure under the traditional model is based on the following assumptions: (1) The number of film prints for each examination item remains constant at the historical average (CT/MR: 1.35 sheets per person; DR: 0.67 sheets per person); (2) The unit purchase price of film remains constant at 16.5 yuan per sheet; (3) The number of patients undergoing examinations matches actual figures. To test the robustness of these assumptions, we conducted a single-factor sensitivity analysis: varying the film print volume by ±10% and the unit price of film by ±10%. The results show that under the most conservative scenario (print volume −10%, unit price −10%), the net savings from the project amounted to 3.472 million yuan; under the most optimistic scenario (print volume +10%, unit price +10%), the net savings amounted to 4.986 million yuan. The actual net savings of 4.1595 million yuan fall within this range, indicating that the conclusions are robust to variations in the assumptions.

#### 3.1.2 Analysis of indirect economic benefits

As of July 31, 2025, the platform has hosted up to 50 TB of imaging data, with an estimated annual increase of approximately 54 TB in the future. The digital cloud imaging service provider offers long-term secure storage and backup for imaging examination data in accordance with storage standards, which significantly alleviates the pressure on hospital local storage servers to expand capacity and reduces operational and maintenance costs.

In addition, with the launch of the digital cloud imaging service platform, radiologic technologists no longer need to format and print films for each patient, reducing the per-person workload by approximately 30 percent—equivalent to a reduction of two technologists (resulting in annual labor cost savings of approximately 240,000 yuan). At the same time, this reduces the costs associated with purchasing and maintaining film printers, yielding annual savings of approximately 150,000 yuan in equipment-related expenses.

### 3.2 Impact on service and efficiency improvements

#### 3.2.1 Significant improvement in physician productivity

Since the launch of the cloud-based imaging platform, the process for radiologists to retrieve and analyze images has been streamlined, and the time spent on non-clinical tasks has been significantly reduced. Clinicians can access the full set of high-resolution DICOM raw images for patients at any time via various devices, including PCs and mobile phones, and the system supports advanced features such as multi-planar reconstruction (MPR) and comparison of historical images. This study extracted data from the PACS system regarding the duration from the completion of a patient’s examination to the completion of image review and report generation by a physician (referred to as “examination-to-report time”) for two time periods: before implementation (January 1, 2022–December 31, 2022) and after implementation (August 12, 2024–August 11, 2025). Since the Shapiro–Wilk normality test indicated that neither dataset followed a normal distribution (*p* < 0.001), the Mann–Whitney *U* nonparametric test was used to compare the differences between the two independent samples.

The comparison of the time from examination to report before and after the project implementation is shown in Table 2. Data analysis revealed that the mean time from examination to report decreased from 15.03 h (standard deviation 57.63 h) before implementation to 6.52 h (standard deviation 48.04 h) after implementation. Since the data exhibited a significant positive skewness, the median and interquartile range were also examined (pre-implementation: median [Q1, Q3] = 6.86 [1.89, 19.65] hours; post-implementation: 2.41 [1.03, 5.69] hours). The *Z*-value from the Mann–Whitney *U* test was −182.39, with *p* < 0.001, indicating a statistically significant difference between the two groups. Based on the median, the time from test to report was reduced by approximately 64.9%.

**Table 2:** Comparison of the time taken to report findings before and after project implementation.

Indicators	Before project implementation (*n* = 160,183)	After the project is implemented (*n* = 202,109)	*Z*	*p*-value
Time from examination to report (h) (mean ± SD)	15.03 ± 57.63	6.52 ± 48.04	−182.39	<0.001
Time from examination to report (h) (Median [Q1, Q3])	6.86[1.89, 19.65]	2.41[1.03, 5.69]		

#### 3.2.2 Comprehensive optimization of the patient experience

After their examinations, patients no longer need to wait in line at the hospital to have their films printed. Instead, they can instantly view their test reports and original DICOM images on their mobile phones by scanning the QR code on their appointment slip or via notifications from the hospital’s official WeChat account, thereby achieving “zero-wait film retrieval.” Figure 5 shows the user interface for the cloud imaging service accessible through the hospital’s official WeChat account and app.

**Figure 5:**
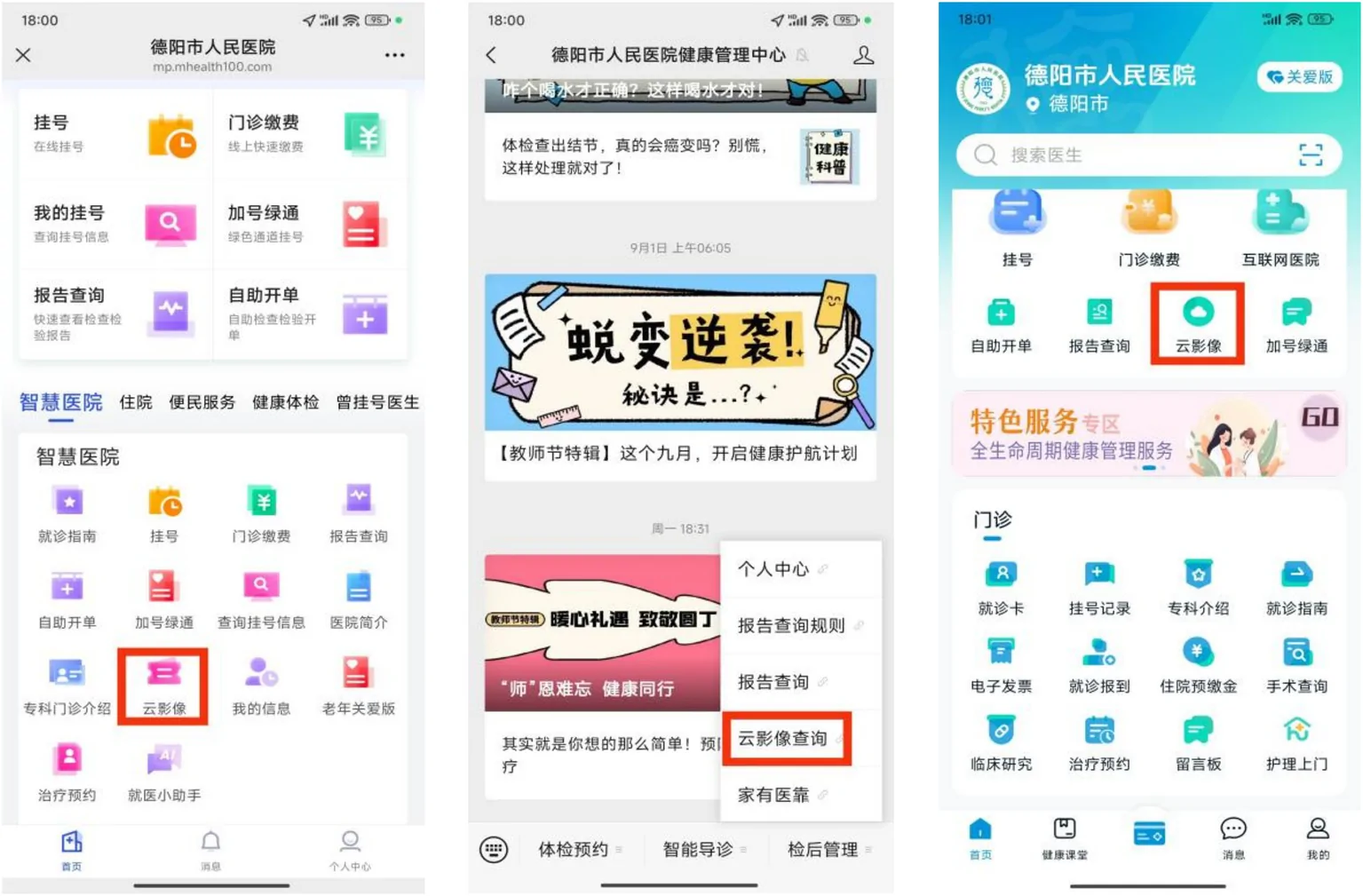
The user interface for accessing the cloud imaging service via the hospital’s official WeChat account and app.

##### 3.2.2.1 Improved patient satisfaction

After the cloud imaging platform went live, the “radiology report turnaround time” metric—as measured by a third-party assessment of patient satisfaction—showed a significant improvement. Prior to the platform’s launch (Q1 2022 through Q2 2024), the average satisfaction rate among outpatient patients regarding radiology report turnaround time was 74.31% (SD = 2.76%); after the launch (Q3 2024 to Q3 2025), the average satisfaction rate was 78.63% (SD = 0.81%). The Cohen’s d effect size for the difference between the two periods was 2.128, indicating a substantial increase in satisfaction.

Interrupted time series analysis. A segmented regression model was used to assess the impact of the cloud imaging system’s launch on satisfaction, and the Newey-West estimator (first-order lag) was employed to address potential autocorrelation and heteroscedasticity. Model fitting results show (Table 3 and Figure 6) that satisfaction exhibited a slight upward trend prior to the intervention (slope β₁ = 0.553, 95% CI: 0.180–0.927, *p* = 0.008). In the first quarter following the system launch, there was an immediate change in the level of satisfaction (β₂ = 0.449, 95% CI: −0.784–1.682, *p* = 0.440), but this change was not statistically significant. The change in the post-intervention slope relative to the pre-intervention slope was β₃ = −0.092 (95% CI: −0.499–0.315, *p* = 0.627), suggesting that the quarterly growth rate of satisfaction did not change significantly after the intervention. However, the post-intervention composite trend (i.e., the sum of the pre-intervention slope and the change in slope) was 0.461 (95% CI: 0.330–0.592, *p* < 0.001), indicating that satisfaction continued to show a significant quarterly upward trend after the system went live. The coefficient of determination (*R*^2^) for the overall model was 0.673.

**Table 3:** Interrupted time series analysis with parameter estimation (OLS + Newey-West standard errors).

Parameters	Estimate	SE	95% CI	*P-*value
Pre-intervention slope	0.553	0.170	0.180, 0.927	0.008
Immediate change in level	0.449	0.560	−0.784, 1.682	0.440
Change in slope	−0.092	0.185	−0.499, 0.315	0.627
Overall Trends Following the Intervention	0.461	0.067	0.330, 0.592	<0.001

**Figure 6:**
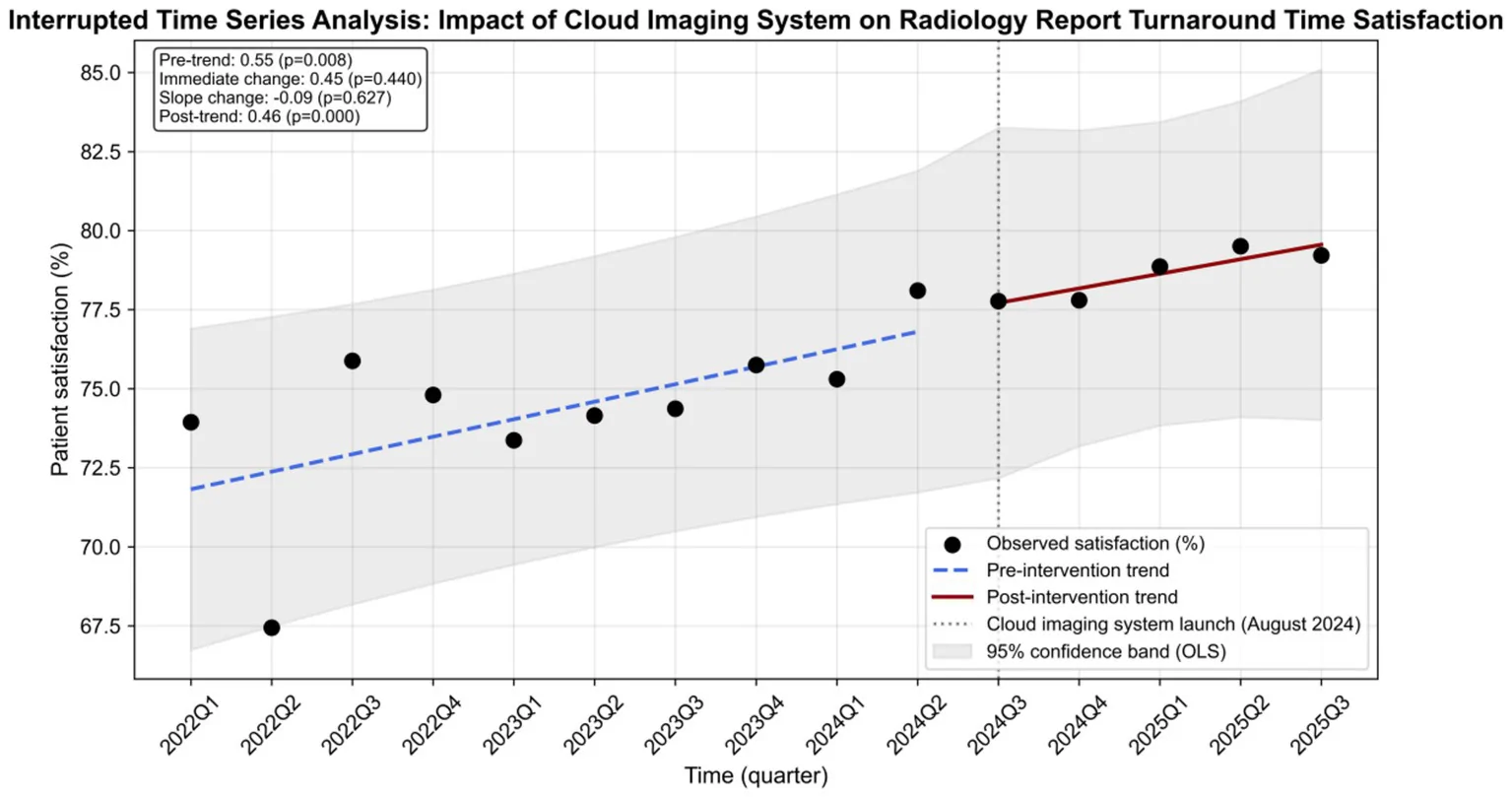
Interrupted time series analysis graph. The data points represent actual satisfaction levels for each quarter; the blue dashed line indicates the fitted trend prior to the intervention, and the red solid line indicates the fitted trend after the intervention; the gray vertical dashed line marks the launch date of the cloud imaging platform (August 2024); the light gray area represents the 95% confidence interval of the OLS forecast.

Residual diagnostics and robustness tests. The autocorrelation function (ACF) and partial autocorrelation function (PACF) plots are shown in Figure 7. The Ljung–Box test for the residuals indicates no significant autocorrelation at lags of 3, 6, and 10 (all *p* > 0.05), and no obvious correlation patterns are observed in the ACF and PACF plots, suggesting that the model specification is reasonable. As part of the robustness analysis, an ARIMA(0,1,1) model incorporating an exogenous intervention variable was further estimated. Although the estimated changes in the level (*β* = 1.536, *p* = 0.954) and slope (*β* = 0.456, *p* = 0.929) from this model did not reach statistical significance, their directions were consistent with those of the main model, and the model residuals passed the white noise test (Ljung–Box Q-test, *p* = 0.22), further supporting the main conclusions of the interrupted time series analysis.

**Figure 7:**
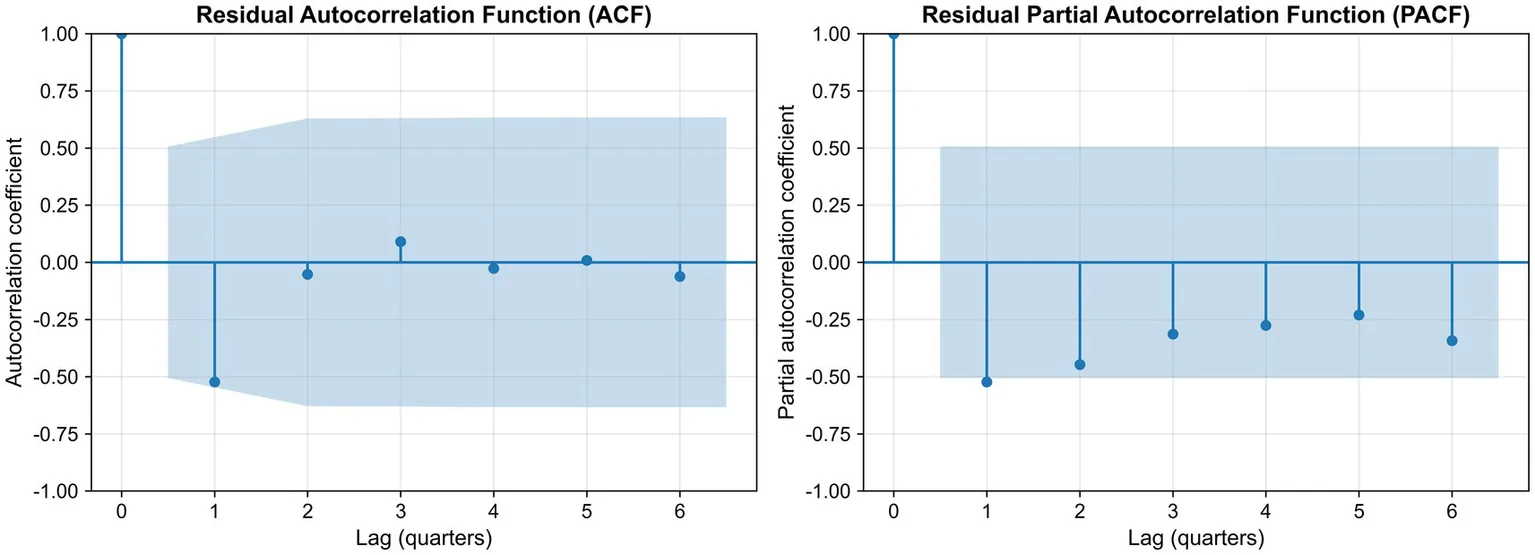
Plots of the autocorrelation function (ACF) and partial autocorrelation function (PACF). The correlation coefficients for all lag orders fall within the 95% confidence interval, indicating that the residuals do not exhibit significant autocorrelation.

In summary, following the launch of the cloud-based imaging platform, patient satisfaction with the time taken to generate radiology reports improved in the short term but did not show a statistically significant jump; however, analysis of long-term trends indicates that satisfaction continued to rise after the intervention, increasing by an average of 0.46 percentage points per quarter, a statistically significant increase (*p* < 0.001).

## 4 Discussion

### 4.1 Key findings and core contributions

The results of this study indicate that a digital cloud-based imaging service platform built using a “service provider upfront investment, pay-for-performance” model has yielded significant benefits in reducing direct consumables expenditures at public hospitals and optimizing imaging service workflows. Within 12 months of the platform’s launch, our center achieved direct consumables cost savings exceeding 4 million yuan, while the median time from examination to reporting was reduced by approximately 64.9%, and patient satisfaction also saw a measurable improvement. These results are consistent with the economic benefits achieved by healthcare institutions worldwide during the transition from traditional film-based to digital imaging workflows: After deploying a clinical information system integrated with RIS/PACS across its network of 40 public hospitals, Mexico’s Social Security Institute (ISSSTE) achieved a complete replacement of radiographic film, significantly reducing operating costs and minimizing the need for repeat examinations (1). Cloud computing architectures can further reduce hospitals’ need for on-premises storage space and specialized operations and maintenance personnel through AI-driven resource optimization strategies (17). A recent review of medical imaging cloud architectures also notes that unified cloud-based imaging platforms can optimize data flows, reduce access latency, and provide clinicians with a standardized portal for accessing images (18).

### 4.2 Business model innovation: the “CAPEX to OPEX” paradigm shift

The core innovation of this study lies in proposing and validating a collaborative mechanism of “upfront investment by the service provider and payment based on performance,” which, at a fundamental level, achieves a paradigm shift in IT infrastructure development from upfront capital expenditures (CAPEX) to on-demand operating expenditures (OPEX). Unlike previous approaches that relied on hospitals to self-fund the construction of on-premises PACS or cloud-based imaging systems (15), this model transfers the initial construction costs and risks associated with technological iteration to the technology service provider, with hospitals paying service fees solely based on actual cost savings. This approach aligns closely with the direction of business model innovation in global healthcare digital transformation (23, 24).

Under this model, the service provider’s revenue is directly linked to service quality and the adoption rate of cloud-based film systems, creating an “incentive-compatible” profit-sharing mechanism. This fundamentally addresses the chronic issue of traditional IT projects prioritizing “construction over operations” and effectively resolves the dilemma faced by some hospitals—where low adoption rates of cloud-based film systems and increased complaints stem from requiring patients to pay additional fees (6).

It must be acknowledged that this study’s argument regarding the advantages of the “pay-for-performance” model is primarily based on conceptual logical reasoning and observational data from a single case; it did not include a control group to directly compare this model with other financing models (such as hospital-built systems or SaaS subscriptions) in terms of cost, efficiency, or sustainability. Therefore, the conclusion that this model is superior to other models should be regarded as an exploratory finding rather than a definitive conclusion. Future research could provide more direct evidence through multicenter, multi-model comparative studies.

### 4.3 Comparison with previous studies

Compared with previous studies, this study expands upon the existing literature in the following ways: First, it systematically quantifies for the first time the direct economic benefits of replacing physical films with cloud-based films (a net savings of 4,159,500 yuan); second, it introduces the ITS quasi-experimental design into the evaluation of cloud-based film effectiveness for the first time, providing more reliable causal evidence than previous descriptive studies (6–8, 15, 16); third, it is the first to report a feasibility study of the “pay-for-performance” model in the field of medical imaging. However, consistent with previous studies, the single-center nature of this study also limits the external validity of its conclusions.

### 4.4 Technical architecture and data security

From a technical implementation perspective, the platform employs a hybrid cloud architecture. Core diagnostic data is stored on local servers to comply with the *Data Security Law* and regulatory requirements for the localization of core medical data, while patient-side image viewing services are deployed on the public cloud, striking a balance between security and convenience. This architecture adheres to the DICOM standard, ensuring interoperability of image data throughout all stages of storage, transmission, and retrieval. The application of emerging standards such as DICOMweb and FHIR further bridges the technical gap between traditional image archiving systems and mobile health applications. This technical design reduces duplicate examinations caused by lost film or inconvenience in carrying it at the source, thereby indirectly conserving regional medical resources.

### 4.5 Social benefits and policy synergy

From a social benefits perspective, the value of this project extends beyond the scope of cost reduction and efficiency improvement at a single hospital. In terms of environmental protection, once the platform goes live, it will reduce the annual use of polyester-based plastic films by hundreds of thousands of sheets, effectively reducing chemical pollution and resource waste caused by silver halide coating waste (1). In terms of regional collaboration, the cloud-based image sharing and regional consultation functions established by the platform provide technical support for the national policy on mutual recognition of examination results (16). At the same time, the cloud-based film information service has significantly reduced film printing and reprint rates, effectively saving healthcare costs and improving patient satisfaction (7, 25). Furthermore, the platform’s capability to retrieve the full set of raw DICOM data lays a high-quality data foundation for subsequent AI-assisted diagnosis, remote multidisciplinary consultations, and clinical research based on real-world data.

### 4.6 Study limitations

This study has the following limitations. First, this study employed a single-center, before-and-after design without a concurrent parallel control group; therefore, it cannot completely rule out the influence of confounding factors—such as seasonal fluctuations in patient volume, concurrent adjustments to other management policies, or natural temporal trends—on the observed results. Although the ITS analysis controlled for observable temporal trends, it cannot rule out the impact on the results of other unobserved reforms that occurred concurrently with the intervention. Second, the observation period was only 12 months. Although the imaging cloud service fee (1.9614 million yuan) during this period covered all operational expenses required for platform operation—including technical support, system maintenance, and cloud storage resources—medium- to long-term risks, such as the costs of long-term technical upgrades, the burden of system maintenance, and the sustainability of third-party service providers’ performance, have not yet been fully assessed. Third, patient satisfaction was derived from the analysis of satisfaction scores related to the imaging examination experience, as reported in the quarterly summary reports of satisfaction surveys commissioned by the hospital from third-party agencies; this carries a certain risk of measurement bias when comparing across studies. Fourth, the satisfaction and efficiency analyses did not adjust for individual-level factors such as patient age, disease severity, and examination type, which may introduce selection bias. Fifth, this study did not conduct a systematic health economic evaluation; key economic indicators—including the cost-effectiveness ratio (CEA), budget impact analysis (BIA), and incremental cost-effectiveness ratio (ICER)—were not calculated. This is primarily because: on the one hand, the calculation of CEA and ICER requires QALYs or clinical outcome measures (such as diagnostic accuracy and the rate of avoided repeat examinations) as the denominator for effectiveness, whereas the data in this study were derived from the hospital’s PACS and financial systems and did not include patient-level long-term health outcome data; on the other hand, BIA requires simulating the impact of this model on health insurance funds or hospital budgets across a larger population or over a longer time frame, which exceeds the scope supported by data from a single-center, 12-month observation period. These limitations restrict a comprehensive assessment of the economic value of this model within a broader healthcare system.

### 4.7 Future research directions

To address the limitations mentioned above, future research could be expanded in the following directions. First, multicenter prospective cohort studies or population-based randomized controlled trials should be conducted, incorporating samples from healthcare institutions at different levels and under different health insurance reimbursement environments, to systematically verify the universality and long-term sustainability of this model. Second, a standardized health economics evaluation framework should be introduced to quantify the economic benefits of this model from both a societal and a healthcare institution perspective, and to conduct a formal cost-effectiveness comparison with the traditional film-based model and the hospital-built cloud PACS model. Third, further focus should be placed on the in-depth mining of the massive DICOM data resources accumulated on the platform—a high-quality imaging database built on the FAIR principles will provide critical infrastructure for the training and validation of AI-assisted diagnostic models. Finally, research on digital transformation in healthcare indicates that the realization of digital technology’s value depends not only on the technology itself but also on synergistic innovation in organizational change and business models (23, 26); therefore, future research should concurrently address transformational factors at the organizational management level.

## 5 Conclusion

Within the framework of this study’s single-center, quasi-experimental design, the digital cloud imaging service platform—built using a “service provider upfront investment, pay-for-performance” model—was found to be temporally associated with reduced film and consumable costs, shorter turnaround times from examination to reporting, and improved patient satisfaction. This model preliminarily demonstrated financial viability over the 12-month observation period and successfully alleviated the prominent issues associated with the traditional plastic film model, such as high costs, low efficiency, and poor service experience.

However, due to limitations in the study design (single-center, before-and-after comparison, and lack of a concurrent control group), further evidence is needed to confirm the causality of the aforementioned associations. The long-term financial sustainability and cross-institutional clinical generalizability of this model require validation through longer-term follow-up and multicenter studies.

The findings of this study suggest that including cloud-based imaging services in medical insurance coverage may be economically justified; however, this conclusion must be based on a more comprehensive national-level health economic evaluation, rather than the single-center data from this study alone.

## Data Availability

The original contributions presented in the study are included in the article/Supplementary material, further inquiries can be directed to the corresponding author.

## Supplementary material

SUPPLEMENTARY TABLE S1 Detailed monthly conversion rate data for cloud imaging from August 12, 2024, to June 15, 2025.

SUPPLEMENTARY TABLE S2 Breakdown of the time from the examination to the completion of the radiology review (time from examination to report issuance).

SUPPLEMENTARY TABLE S3 Data on the “time to receive radiology reports” indicator from an outpatient satisfaction survey conducted by a third-party professional firm.
